# The Emergence of Miller's Magic Number on a Sparse Distributed Memory

**DOI:** 10.1371/journal.pone.0015592

**Published:** 2011-01-05

**Authors:** Alexandre Linhares, Daniel M. Chada, Christian N. Aranha

**Affiliations:** 1 Getulio Vargas Foundation/EBAPE, Rio de Janeiro, Brazil; 2 Cortex Intelligence/R. Assembleia 10, Rio de Janeiro, Brazil; Indiana University, United States of America

## Abstract

Human memory is limited in the number of items held in one's mind—a limit known as “Miller's magic number”. We study the emergence of such limits as a result of the statistics of large bitvectors used to represent items in memory, given two postulates: i) the Sparse Distributed Memory; and ii) chunking through averaging. Potential implications for theoretical neuroscience are discussed.

## Introduction

Human short-term memory is severely limited. While the existence of such limits is undisputed, there is ample debate concerning their nature. Miller [1] described the ability to increase storage capacity by grouping items, or “chunking”. He argued that the span of attention could comprehend somewhere around seven information items. Chunk structure is recursive; as chunks may contain other chunks as items: Paragraphs built out of phrases built out of words built out of letters built out of strokes. This mechanism is used to explain the cognitive capacity to store a seemingly endless flux of incoming, pre-registered, information, while remaining unable to absorb and process new (non-registered) information in highly parallel fashion.

Miller's ‘magic number seven’ has been subject of much debate over the decades. Some cognitive scientists have modeled such limits by simply using (computer-science) “pointers”, or “slots” (e.g, [2], [3]—see [4], [5] for debate). However, such approaches do not seem plausible given the massively parallel nature of the brain, and we believe memory limits are an emergent property of the neural architecture of the human brain. As Hofstadter put it a quarter of a century ago [6] : the “problem with this [slot] approach is that it takes something that clearly is a very complex consequence of underlying mechanisms and simply plugs it in a complex structure, bypassing the question of what those underlying mechanisms might be.”(p. 642)

Our objective in this paper is to study these memory limits as emergent effects of underlying mechanisms. We postulate two mechanisms previously discussed in the literature. The first is a mathematical model of human memory brought forth by Kanerva [7], called Sparse Distributed Memory (SDM). We also presuppose, following [8], an underlying mechanism of chunking through averaging. It is not within the scope of this study to argue for the validity of SDM as a cognitive model; for incursions on this broader topic, we refer readers to [9]–[11], which discuss the plausibility of this Vector Symbolic Architecture family of models (in which SDM is contained).

This work, while similar in its mathematical foundations, is different from previous capacity analyses: In [7], the memory capacity analysis of SDM relates to its long-term memory mechanisms, while we study its short–term memory limits. Our work also differs from that of Plate, in that, regardless of the number of items presented, the memory will only store (and subsequently retrieve) a psychologically plausible number of items. The difference becomes salient in Plate's own description [12]: “As more items and bindings are stored in a single HRR the noise on extracted items increases. If too many associations are stored, the quality will be so low that the extracted items will be easily confused with similar items or, in extreme cases, completely unrecognizable”(p. 139). Plate is focused on long–term memory; and we will focus on Miller's STM limits.

A number of theoretical observations are drawn from our computations: i) a range of plausible numbers for the dimensions of the memory, ii) a minimization of a current controversy between different ‘magic number’ estimates, and iii) potential empirical tests of the chunking through averaging assumption. We should start with a brief description of our postulates: i) the SDM, and ii) chunking through averaging.

### Sparse Distributed Memory

The Sparse Distributed Memory (SDM), developed in [7], defines a memory model in which data is stored in distributed fashion in a vast, sparsely populated, binary address space. In this model, (a number of) neurons act as *address decoders*. Consider the space 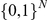: SDM's address space is defined allowing 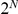 possible locations, where 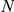 defines both the word length and the number of dimensions of the space: the memory holds binary vectors of length 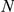. In SDM, the data is the same as the medium in which it is stored (i.e. the stored items are 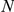-bit vectors in 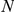-dimensional binary addresses).

SDM uses Hamming distance as a metric between any two 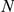-bit vectors (hereafter memory items, items, elements, or bitstrings—according to context). Neurons, or *hard locations* (see below), in Kanerva's model, hold random bitstrings with equal probability of 0's and 1's—Kanerva [13], [14] has been exploring a variation of this model with a very large number of dimensions (around 10000). (With the purpose of encoding concepts at many levels, the Binary Spatter Code—or BSC—, shares numerous properties with SDM.) By using the Hamming distance as a metric, one can readily see that the average distance between any two points in the space is given by the binomial distribution, and approximated by a normal curve with mean at 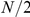 with standard deviation 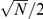. Given the Hamming distance, and large 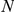, most of the space lies close to the mean. A low Hamming distance between any two items means that these memory items are associated. A distance that is close to the mean 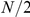 means that the memory items are orthogonal to each other. This reflects two facts about the organization of human memory: i) *orthogonality of random concepts*, and ii) *close paths between random concepts*.

*Orthogonality of random concepts*: the vast majority of concepts is orthogonal to all others. Consider a non-scientific survey during a cognitive science seminar, where students asked to mention ideas unrelated to the course brought up terms like *birthdays*, *boots*, *dinosaurs*, *fever*, *executive order*, *x-rays*, and so on. Not only are the items unrelated to cognitive science, the topic of the seminar, but they are also unrelated to each other.

*Close paths between concepts*: The organization of concepts seems to present a ‘small world’ topology–for an empirical approach on words, for instance, see [15]. For any two memory items, one can readily find a stream of thought relating two such items (“Darwin gave *dinosaurs* the *boot*”; “she ran a *fever* on her *birthday*”; “isn't it time for the Supreme Court to *x-ray* that *executive order*?” …and so forth). Robert French presents an intriguing example in which one suddenly creates a representation linking the otherwise unrelated concepts of “coffee cups” and “old elephants” [16]. In sparse distributed memory, any two bitstrings with Hamming distance around 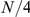 would be extremely close, given the aforementioned distribution. And 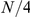 is the expected distance of an average point between two random bitstrings.

Of course, for large 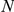 (such as 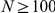), it is impossible to store all (or even most) of the space—the universe is estimated to carry a storage capacity of 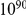 bits (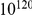 bits if one considers quantum gravity) [17]. It is here that Kanerva's insights concerning sparseness and distributed storage and retrieval come into play: 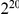
—or a number around one million—physical memory locations, called hard locations, could enable the representation of a large number of different bitstrings. Items of a large space with, say, 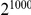 locations would be stored in a mere 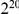 hard locations—the memory is indeed sparse.

In this model, every single item is stored in several hard locations, and can, likewise, be retrieved in distributed fashion. Storage occurs by distributing the item in every hard location within a certain threshold ‘radius’ given by the Hamming distance between the item's address and the associated hard locations. Different threshold values for different numbers of dimensions are used (in his examples, Kanerva used 100, 1000 and 10000 dimensions). For 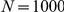, the distance from a random point of the space to its nearest (out of the one million) hard locations will be approximately 424 bits [7] (p.56). In this scenario, a threshold radius of 451 bits will define an *access sphere* containing around 1000 hard locations. In other words, from any point of the space, approximately 1000 hard locations lie within a 451-bit distance. All of these accessible hard locations will be used in storing and retrieving items from memory. We therefore define the function 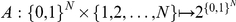 and a hard location 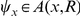 iff 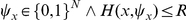, where 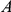 defines an access radius around 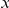 of size 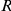 (451 if 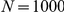; 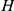 is the Hamming distance).

A brief example of a storage and retrieval procedure in SDM is in order: to store an item 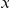 at a given (virtual) location 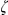 (in sparse memory) one must activate every hard location within the access sphere of 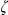 (see below) and store the datum in each one. Hard locations carry 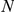 adders, one for each dimension. To store a bitstring 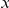 at a hard location 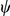, one must iterate through the adders of 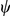: If the 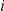-th bit of 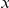 is 1, increment the 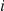-th adder of 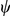, if it is 0, decrement it. Repeating this for all hard locations in 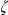
's access sphere will distribute the information in 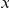 throughout these hard locations.

Retrieval of data in SDM is also massively collective and distributed: to peek the contents of each hard location, one computes its related bit vector from its adders, assigning the 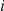-th bit of 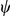 as a 1 or 0 if the 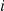-th adder is positive or negative, respectively (a coin is flipped if it is 0). Notice, however, that this information in itself is meaningless and may not correspond to any one specific datum previously registered. To read from a location 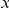 in the 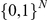 address space, one must activate the hard locations in the access sphere of 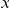 and gather each related bit vector. The stored datum will be the majority rule decision of all activated hard locations' related bit vectors. If, for the 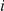-th bit, the majority of all bit vectors is 1, the final read datum's 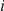-th bit is set to 1, otherwise to 0. Thus, “SDM is distributed in that many hard locations participate in storing and retrieving each datum, and one hard location can be involved in the storage and retrieval of many data” [18] (p. 342).

All hard locations within an access radius collectively point to an address. Note also that this process is iterative. The address obtained may not have information stored on it, but it provides a new access radius to (possibly) converge to the desired original address. One particularly impressive characteristic of the model is its ability to simulate the “tip-of-tongue” phenomenon, in which one is certain about some features of the desired memory item, yet has difficulty in retrieving it (sometimes being unable to do so). If the requested address is far enough from the original item (209 bits if 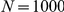), iterations of the process will not decrease the distance—and time to convergence goes to infinity.

The model is robust against errors for at least two reasons: i) the contribution of any one hard location, in isolation, is negligible, and ii) the system can readily deal with incomplete information and still converge to a previously registered memory item. The model's sparse nature dictates that any point of the space may be used as a storage address, whether or not it corresponds to a hard location. By using about one million hard locations, the memory's distributed nature can “virtualize” the large address space. The distributed aspect of the model allows such a virtualization. Kanerva [7] also discusses the biological plausibility of the model, as the linear threshold function given by the access radius can be readily computed by neurons, and he suggests the interpretation of some particular types of neurons as address decoders. Given these preliminaries concerning the Sparse Distributed Memory, we should now proceed to our second premise: *chunking through averaging*.

### Chunking through averaging

To chunk items, the majority rule is applied to each bit: given 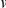 bitstrings to be chunked, for each of the 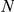 bits, if the majority is 1, the resulting bitstring's chunk bit is set to 1; otherwise it is 0. In case of perfect ties (no majority), a coin is flipped.

We have chosen the term ‘chunking’ to describe an averaging operation, and ‘chunk’ to describe the resulting bitstring, because, through this operation, the original components generate a new one to be written to memory. The reader should note, in SDM's family of high-dimensional vector models, called Vector Symbolic Architectures (VSA), the operation that generates composite structures is commonly known as superposition [10]–[12].

Obviously, this new chunked bitstring may be closer, in terms of Hamming distance, to the original elements, than the mean distance 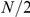 between random elements (500 bits if 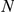 = 1000), given a relatively small 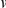. The chunk may then be stored in the memory, and it may be used in future chunking operations, allowing, thus, for recursive behavior. With these preliminaries, we turn to numerical results in the analysis section.

## Analysis

### Computing the Hamming distance from a chunk 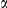 to items

Let 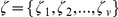 be the set of bitstrings to be chunked into a new bitstring, 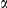. The first task is to find out how the Hamming distance is distributed between this averaged 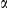 bitstring and the set 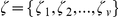 of bitstrings being chunked. This is, as discussed, accomplished through majority rule at each bit position. Imagine that, for each separate dimension, a supreme court will cast a decision with each judge choosing yes (1) or no (0). If there is an even number of judges, a fair coin will be flipped in the case of a tie. Given that there are 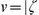 votes cast, how many of these votes will fall in the minority side? (Each minority-side vote adds to the Hamming distance between an item 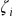 and the average 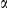.)

Note that the minimum possible number of *minority votes* is one, and that it may occur with either 3 votes cast or two votes and a coin flip. If there are two minority votes, they may stem from either 5 votes or 4 votes and a coin flip, and so forth. We thus have that, for 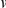 votes, the maximum minority number is given by 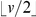 (and the ambiguities between an odd number of votes versus an even number of votes plus a coin flip are resolved by considering 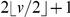 total votes). This leads to independent Bernoulli trials, with success factor 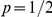, and the constraint that the minority view differs from the majority bit vote. Let 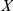 be a random variable with the number of minority votes. Obviously in this case, 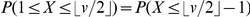, hence we have, for 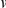 items, the following cumulative distribution function of minority votes [19]:
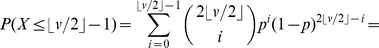


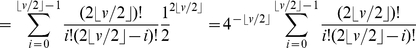



While we can now, given 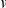 votes, compute the distribution of minority votes, the objective is not to understand the behavior of these minority bits *in isolation*, i.e., per dimension on the chunking process. We want to compute the number of dimensions to (in a psychologically and neurologically plausible way) store and retrieve around 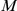 items—Miller's number of retrievable elements—through an averaging operation. Hence we need to compute the following:

- Given a number of dimensions 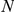 and a set 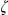 of items, the probability density function of the Hamming distance from 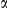 to the chunked elements 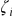,
- A threshold 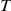: a number of dimensions in which, if an element 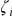
's Hamming distance to 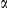 is farther from that point, then 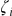 cannot be retrieved,
- As 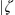 grows, how many elements remain retrievable?

Given bitstrings with dimension 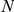, suppose 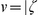 elements have been chunked, generating a new bitstring 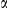. Let 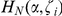 be the Hamming distance from the chunked element 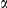 to 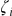, the 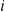-th element of 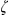. What is the distance from 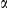 to elements in 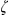
? Here we are led to 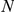 Bernoulli trials with success factor 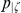. Since 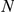 is large, 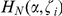 for 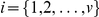 can be approximated by a Normal distribution, we may use 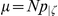 and 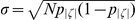. To model human short term memory's limitations, we want to compute a cutoff threshold 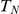 which will guarantee retrieval of around 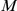 items averaged in 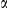 and “forget” an item 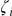 if 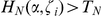
—where 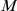 is Miller's limiting number. Hence to guarantee retrieval of around 95% (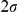) of 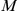 items, we have 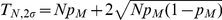, where 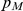 is the success factor corresponding to 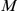. Note that Cowan [20] has argued for a “magic number” estimate of 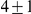 items—and the exact cognitive limit is still a matter of debate. The success factor for 4 (or 5) elements is 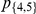 = .3125; and for 6 (or 7) elements it is 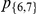 = .34375. By fixing the success factor at plausible values of 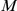 (at {4,5}, or at an intermediary value between {4,5} and {6,7}, or at {6,7}), different threshold values 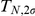 are obtained for varying 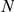, as shown in Table 1. In the remainder of this study, we use the intermediary success factor 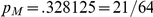 for our computations; again without loss of generality between different estimates of 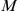.

**Table 1:** Threshold values.

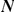	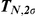 ( 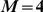 or 5)	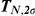 (intermediary value)	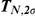 ( 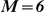 or 7)
64	27.42	28.51	29.6
128	50.49	52.62	54.75
192	72.85	76.01	79.16
256	94.83	99.02	103.2
320	116.58	121.8	126.99
384	138.17	144.4	150.61
448	159.62	166.88	174.11
512	180.98	189.25	197.49
576	202.25	211.54	220.8
640	223.45	233.76	244.03
704	244.6	255.92	267.2
768	265.69	278.02	290.32
832	286.74	300.09	313.4
896	307.75	322.11	336.43
960	328.72	344.1	359.43
1024	349.66	366.05	382.4
100	40.52	42.2	43.87
1000	341.82	357.82	373.79
10000	3217.7	3375.16	3532.49

Thresholds 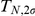 given plausible success factors and dimension combinations.

We thus have a number of plausible thresholds and dimensions. We can now proceed to compute the plausibility range: Despite the implicit suggestion in Table 1 that any number of dimensions might be plausible, how does the behavior of these 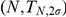 combinations vary as a function of the number of presented elements, 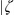
?

### Varying the number of presented items

Consider the case of information overload, when one is presented with a large set of items. Suppose one were faced with dozens, or hundreds, of distinct items. It is not psychologically plausible that a large number of elements should be retrievable. For an item 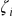 to be impossible to retrieve, the distance between the averaged item 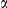 and 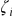 must be higher than the threshold point of the corresponding 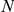. When we have an increasingly large set of presented items, there will be information loss in the chunking mechanism, but it should still be possible to retrieve some elements within plausible psychological bounds.

**Figure 1:**
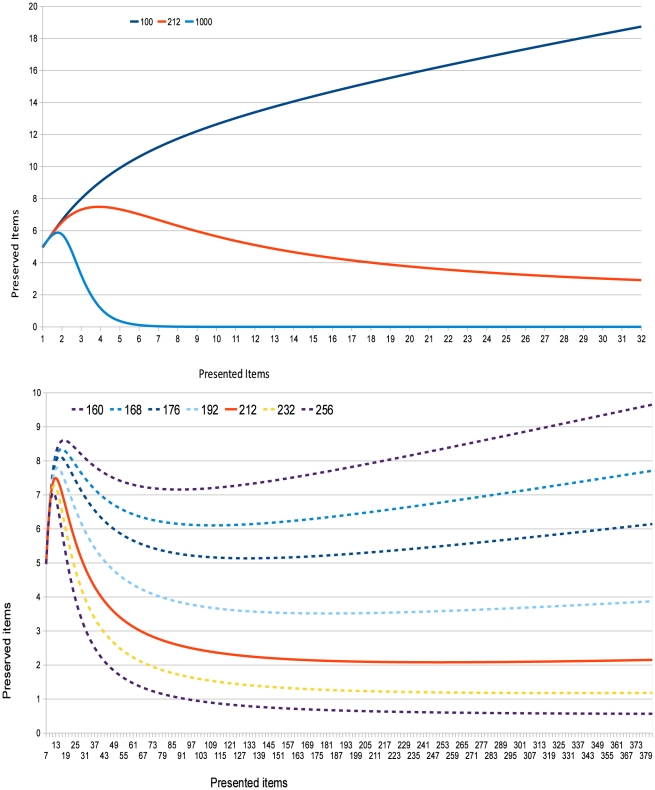
Preserved items as a function of 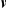; selected values of 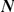.

Figure 1(a) shows the behavior of three representative sizes of 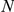: 100, 212 and 1000 dimensions. (100 and 1000 were chosen because these are described in Kanerva's original examples of SDM.) 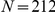 has shown to be the most plausible number of dimensions, preserving a psychologically plausible number of items after presentations of different set sizes. It is clear that 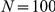 quickly diverges, retaining a high number of items in a chunk (as the number of presented items grows). Conversely, if 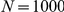, the number of preserved memory items rapidly drops to zero, and the postulated mechanisms are unable to retrieve any items at all—a psychologically implausible development. Figure 1(b) zooms in to illustrate behavior over a narrower range of 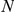-values and a wider range of presented items. Varying the number of presented items and computing the number of preserved items (for a number of representative dimensions) yields informative results. Based on our premises, experiments show that to appropriately reflect the storage capacity limits exhibited by humans, certain ranges of 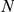 must be discarded. With too small a number of dimensions, the model will retrieve too many items in a chunk. With too large a number of dimensions, the model will retrieve at most one or two—perhaps no items at all. This is because of the higher number of standard deviations involved in the dimension sizes: for 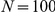, the whole space has 20 standard deviations, and 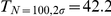 is less than 2 standard deviations below the mean—which explains why an ever growing number of items is “retrieved” (i.e., high probability of false positives). For 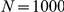, the space has over 63 standard deviations, and 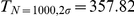, is around 8.99 standard deviations *below* the mean. There is such a minute part of the space below 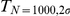 that item retrieval is virtually impossible.

With an intermediary success factor 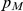 between 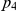 and 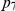 established by the cognitive limits 4 and 7, we have computed the number of dimensions of a SDM as lying in the vicinity of 212 dimensions. Variance is minimized when 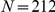
—and retrieval results hold psychologically plausible ranges even when hundreds of items are presented (i.e., the SDM would be able to retrieve from a chunk no more than nine items and at least one or two, regardless of how many items are presented simultaneously). Finally, given that this work rests upon the chunking through averaging postulate, in the next section we will argue that the postulated mechanism is not only plausible, but also empirically testable.

## Results and Discussion

### The chunking through averaging postulate

Consider the assumption of chunking through averaging. We propose that it is plausible and worthy of further investigation, for three reasons.

First, it minimizes the current controversy between Miller's estimations and Cowan's. The disparity between Miller's 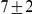 or Cowan's 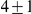 observed limits may be a smaller delta than what is argued by Cowan. Our “chunking-through-averaging” premise may provide a simpler, and perhaps unifying, position to this debate. If chunking 4 items has the same probability as 5 items, and chunking 6 items is equivalent to chunking 7 items, one may find that the ‘magic number’ constitutes one cumulative probability degree (say, 4-or-5 items) plus or minus one (6-or-7 items).

A mainstream interpretation of the above phenomenon may be that, as with any model, SDM is a simplification; an idealized approximation of a presumed reality. Thus, one may see it as insufficiently complete to accurately replicate the details of true biological function due to, among other phenomena, inherent noise and spiking neural activity. In this case, one would interpret it as a weakness, or an inaccuracy inherent to the model. An alternative view, however improbable, may be that the model is accurate in this particular aspect, in which case, the assumption minimizes the current controversy between Miller's estimations and Cowan's.

The success factors computed above show that for either 4 or 5 items, we have 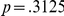, while for 6 or 7 items we have 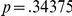. If we assume an intermediary value of 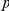
—which is reasonable, due to noise or lack of synchronicity in neural processing—the controversy vanishes. We chose to base our experiments on the mean value (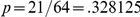), and the results herein may be adapted to other estimates as additional experiments settle the debate.

Moreover, a chunk 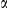 tends to be closer to the 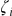 chunked items than these items are between themselves. For example, with 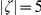 and 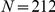, the Hamming distance between a chunk and a random item is drawn from a distribution with 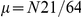 and 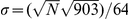; in here, from the point of view of the chunked item 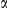, the closest 1% of the space lies at 53 bits, while 99% of the space lies at 84 bits. Contrast this with the distances between any two random, orthogonal, items, which are drawn from 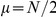 and 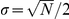: from the point of view of a random item, the closest 1% of the space lies at 89 bits, while 99% of the space lies at 122. This disparity reflects the principles of *orthogonality between random concepts* and of *close paths between concepts* (or small worlds [15]): the distance between 2 items from any 5 is large, but the distance to the average of the set is small. Of course, as 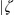 grows, the distance to 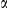 also grows (since 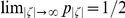), and items become irretrievable. One thing is clear: with 5 chunked items, the chance of retrieving a false positive is minute.

Finally, the assumption of chunking through averaging is empirically testable. Psychological experiments concerning the difference in ability to retain items could test this postulate. The assumption predicts that (4, 5) items, or more generally that (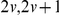) for integer 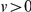 will be registered with equal probability. It also predicts how the probability of 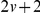 retained items should drop in relation to 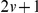 if 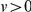. This is counterintuitive and can be measured experimentally. Note, however, two qualifications: first, as chunks are hierarchically organized, these effects may be hard to perceive in experimental settings. One would have to devise an experimental setting with assurances that only chunks from the same level are retrievable–neither combinations of such chunks, nor combinations of their constituting parts. The final qualification is that, as 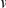 grows, the aforementioned probability difference tends to zero. Because of the conjunction of these qualifications, this effect would be hard to perceive on normal human behavior.

### Concluding remarks

Numerous cognitive scientists model the limits of human short-term memory through explicit “pointers” or “slots”. In this paper we have considered the consequences of a short-term memory limit given the mechanisms of i) Kanerva's Sparse Distributed Memory, and ii) chunking through averaging. Given an appropriate choice for the number of dimensions of the binary space, we are able to model chunks that limit active memory's storage capacity, while allowing the theoretically endless recursive association of pre-registered memory items at different levels of abstraction (i.e., chunks may be chunked with other chunks or items, indiscriminately [1], [21]). This has been pointed out in [22], however, in here we use the short-term memory limitations as a bounding factor to compute plausible ranges for 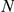.

Some observations are noteworthy. First, our work provides plausible bounds on the number of dimensions of a SDM—we make no claims concerning Kanerva's recent work (e.g., [14]). Given our postulates, it seems that 100 dimensions is too low a number, and 1000 dimensions too high. In our computations, assuming 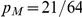, variance of the number of items retained (as a function of the number of presented items and at least one retrievable item) was minimized at 212 dimensions. This value was chosen as our optimal point of focus for it provided stable, psychologically plausible behavior for a wide range of set sizes. We have concentrated on the SDM and chunking through averaging postulates, yet future research could also look at alternative neural models; for it is certain that the brain does not use explicit slots or pointers when items are chunked. One can reasonably argue: what good can come from replacing one magic number with another? There are two potential benefits: first, by fixing parameter 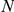, we can restrict the design space of SDM simulations and ensure that a psychologically plausible number of items is chunked. Another advantage is theoretical: the number 212 suggests that we should look for neurons that seem to have, or respond majoritarily to, such a number of active inputs in their linear threshold function.

Of course, a single 212 bit vector in SDM does not encode meaningful content at all. The existence of a bitstring can only be meaningful in relation to other bitstrings close to it. Consider, for instance, an A4 sheet of paper, of size 210mm×297mm (8.3in×11.7in). A 1200×1200 dots-per-inch printer holds less than 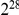 potential dots in an entire sheet. While the space of possible black and white printed A4 sheets is a very large set of 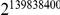 possible pages, the vast majority of them, rather like the library of Babel, are composed of utter gibberish. Any single dot needs only 28 bits to be described, and because the dots usually cluster into strokes, chunks can be formed. Moreover, because strokes cluster to form fonts, which cluster to form words, which cluster to form phrases and paragraphs; combinations of large sets of 212 dimensional bitstrings can encode the meaningful content of pages and books—provided those items have been previously chunked in the reader's mind. Without chunks there can be no meaning; this paragraph, translated to Yanomami (assuming that's possible), would become unreadable to its intended audience and to its authors.

Sparse Distributed Memory holds a number of biologically and psychologically plausible characteristics. It is associative, allowing for accurate retrieval given vague or incomplete information (which is relevant given the potential for asynchronous behavior [23]); it is readily computable by neurons; it seems suitable for storage and retrieval of low-level sensorimotor information [24], it is a plausible model of the space of human concepts, and it exhibits a phenomenon strikingly similar to the tip-of-the-tongue situation. With the results presented herein, sparse distributed memory also reflects the natural limits of human short-term memory.
